# The impact of secondary forest regeneration on ground-dwelling ant communities in the Tropical Andes

**DOI:** 10.1007/s00442-019-04497-8

**Published:** 2019-09-04

**Authors:** Matthew G. Hethcoat, Bethany J. King, Fernando Fernandez Castiblanco, Claudia M. Ortiz-Sepúlveda, Fabian Camilo Prada Achiardi, Felicity A. Edwards, Claudia Medina, James J. Gilroy, Torbjørn Haugaasen, David P. Edwards

**Affiliations:** 1 School of Mathematics and Statistics, University of Sheffield, Sheffield, S3 7RH UK; 2 Department of Animal and Plant Sciences, University of Sheffield, Sheffield, S10 2TN UK; 3 Instituto de Ciencias Naturales Universidad Nacional, Carrera 30#45-03, Bogota, Colombia; 4 UMR 8198–Evolution, Ecologie et Paléontologie, CNRS, Université de Lille, 59000 Lille, France; 5 Instituto de Investigacion de Recursos Biologicos Alexander von Humboldt, Calle 28A#15-09 Bogota, Colombia; 6 School of Environmental Science, University of East Anglia, Norwich, NR4 7TJ UK; 7 Faculty of Environmental Sciences and Natural Resource Management, Norwegian University of Life Sciences, Ås, Norway

**Keywords:** Cattle pasture, Cloud forest, Formicidae, Surface-active ants, Natural regeneration, Secondary forest

## Abstract

Natural regeneration of abandoned farmland provides an important opportunity to contribute to global reforestation targets, including the Bonn Challenge. Of particular importance are the montane tropics, where a long history of farming, frequently on marginal soils, has rendered many ecosystems highly degraded and hotspots of extinction risk. Ants play crucial roles in ecosystem functioning, and a key question is how time since abandonment and elevation (and inherent temperature gradients therein) affect patterns of ant recovery within secondary forest systems. Focusing on the Colombian Andes across a 1300 m altitudinal gradient and secondary forest (2–30 years) recovering on abandoned cattle pastures, we find that over time ant community composition and species richness recovered towards that of primary forest. However, these relationships are strongly dependent on elevation with the more open and warmer pasturelands supporting more ants than either primary or secondary forest at a particular elevation. The loss of species richness and change in species composition with elevation is less severe in pasture than forests, suggesting that conditions within pasture and its remaining scattered trees, hedgerows and forest fragments, are more favourable for some species, which are likely in or near thermal debt. Promoting and protecting natural regenerating forests over the long term in the montane tropics will likely offer significant potential for returning ant communities towards primary forest levels.

**Electronic supplementary material:**

The online version of this article (10.1007/s00442-019-04497-8) contains supplementary material, which is available to authorized users.

## Introduction

There is an emerging global agenda of forest recovery, to reduce the rate of climate change, to recover biodiversity, and in turn, to return the provision of ecosystem services and rural livelihoods to degraded landscapes (Lamb et al. 2005). The 2011 Bonn Challenge aims to restore 350 million hectares of degraded forest landscapes by 2030 to avoid a global temperature increase of > 2 °C (UNFCCC 2015) whilst helping to meet the Sustainable Development Goals. In addition, Target 15 of the Aichi targets aims to restore 15% of degraded ecosystems by 2020, with a focus on biodiversity and ecosystem restoration (Swinfield et al. 2016).

Of particular importance in the restoration agenda are the montane tropics, where a long history of farming, frequently on marginal soils, has rendered many ecosystems highly degraded, with little remaining natural habitat. The montane tropics are also some of the ecosystems with highest global endemicity, making them hotspots of extinction risk. In the northern Tropical Andes, for example, there is a hyperdiversity of endemic species, yet 75% of natural habitat has been converted, largely to low-intensity cattle pasture (Gilroy et al. 2014a, b). In many montane regions, there is an on-going phase of farmland abandonment via rural–urban migration, in part due to the poor profitability of farming in marginal areas. For example, between 2001 and 2010, 36 million ha of land was naturally regenerated throughout Latin America and the Caribbean (Aide et al. 2013), with much of this centred at higher elevations (Nanni et al. 2019). Naturally regenerating secondary forests will thus play a critical role in reaching global restoration targets, because they provide a cheap, large-scale and minimum effort solution to reforestation (Gilroy et al. 2014a, b) and because funds for conservation projects are limited (McCarthy et al. 2012).

Despite the crucial role of ants in ecosystem functioning (Bihn et al. 2010), as environmental bioindicators (Chen et al. 2011; Agosti et al. 2000) and as model taxa for understanding the impacts of land-use change in other groups (Edwards et al. 2014; Stork et al. 2017), our review of the literature revealed only 23 studies on the impacts of regenerating secondary forest relative to other land uses on ants. Critically, only three of these studies focused on the montane tropics, including hyperdiverse cloud forests (Schonberg et al. 2004; Chen et al. 2011; Tiede et al. 2017), where much restoration is likely to occur. In Southwest China, where scale insects are promoted to produce harvestable lac resin, lac plantations had higher species richness of ground- and canopy-dwelling ants than did secondary forest (Chen et al. 2011). Similarly in Costa Rica, secondary forests had lower species richness of arboreal ants than primary forests and pastures, driven by a lack of rich epiphyte mats in secondary forest tree that were still present in primary forests and relict trees within pastures (Schonberg et al. 2004). In Ecuador, there was no difference in ant occurrence, species and functional richness between primary and secondary forest sites (Tiede et al. 2017). The low number of studies and inconsistent relationships highlight core knowledge gaps in our understanding of the potential for tropical montane secondary forests to harbour ants.

Studies are needed in the montane tropics that sample across a range of secondary forest ages, because we might expect communities to become more similar to those in primary forest over time as forest structure/complexity recovers (Klimes et al. 2012) and ants recolonise, with such a pattern occurring in other taxa (e.g. montane birds and dung beetles; Gilroy et al. 2014a, b; Edwards et al. 2017). In addition, we may expect communities to shift in secondary forest over time from those found in pastures as habitat structure, canopy cover, and the thermal environment is transitioning (e.g. González del Pliego et al. 2016). Additionally, we need to further investigate the pasture system beyond tree crowns, to better understand the ground-dwelling any communities (Schonberg et al. 2004). None of the 23 studies (Table S1) explicitly investigated the effect of elevation (and inherent temperature gradients therein) on patterns of ant diversity between secondary and primary forests, yet prior works have shown negative relationships between elevation and ant diversity (Araújo and Fernandes 2003, Brazil; van Ingen et al. 2008, Australia; Staab et al. 2014, China; Tiede et al. 2017, Ecuador). Finally, we need work based in the montane Neotropics generally, an important hotspot of ant diversity (Guénard et al. 2012).

In this study, we use ground-dwelling ant communities as bioindicators to examine whether naturally regenerated secondary forest across an elevational gradient in the montane tropics deliver biodiversity recovery, and thus infer recovery of core ecosystem functions. Ants are good bioindicators, being extremely abundant, an ecologically important group involved in several positive and negative biological interactions, and as providers of key ecosystem functions, including seed dispersal, control of invertebrate communities through predation, and bioturbation of the soil. We focus on the Tropical Andes hotspot of biodiversity, along a landscape transition from cattle pasture, through maturing secondary forest and primary forests that span over 1300 m in elevation. In doing so, we provide the first assessment of the value of secondary forests for ants in the Tropical Andes biodiversity hotspot, whilst also accounting for the as-yet overlooked, but potentially confounding, impact of elevation on patterns observed.

## Materials and methods

### Study location

The study was conducted in the western Colombian Andes, South America, within the departments of Antioquia, Risaralda and Chocó (approximately 100 km southwest of Medellín), which all straddle the historic agricultural frontier (Online Resource Fig. S1; 5.71 N, − 76.04 W). The study area spans an elevational range of 1351–2682 m above sea level characterised by subtropical montane rainforest to montane cloud forest and widespread cattle farming. The study sites comprised three broad habitat types: primary forest, naturally regenerating secondary forests (with a range of 7–30 years old) and an agricultural matrix dominated by pasture. Within the study areas, pasture habitats typically included some primary and secondary forest fragments, isolated trees and hedgerows, as well as a limited cover (< 10 ha in total) of other crops. Young secondary forests (defined as < 15 years, Gilroy et al. 2014a, b) were all owned by conservation NGOs who provided stand ages through detailed records. Ages of mature secondary forest (defined as > 15 years) were obtained through informal interviews with reserve managers and locals, with mean ages used where differing. All secondary forests were unmanaged, expect for cattle exclusion, and had some degree of connectivity to primary forest. For more details see Gilroy et al. (2014a, b).

### Ant sampling

Sampling took place between July and August in 2014, which corresponded with the relatively dry period in the region. Ant communities were sampled within 36 squares of 400 × 400 m, located across three study landscapes and spanning all habitat types (Online Resource Fig. 1; following Gilroy et al. 2014a, b). Thirty-two squares contained one habitat type (11 primary, 9 secondary, and 12 pasture) and were spaced ≥ 300 m apart between habitats and ≥ 400 m apart within habitats. The remaining six squares were mixed and contained two habitats. Ants were collected using pitfall traps (250-ml plastic cups embedded within the soil) without bait and filled with a little water containing a few drops of odourless soap to break the surface tension (Lopes and Vasconcelos 2008). Pitfalls were activated immediately after placement in the soil.

Within each square, we placed five pitfall traps with minimum 100 m spacing to ensure community independence (Woodcock et al. 2011). This approach samples ants in a relatively small radius around the pitfall trap; for example, in Borneo, most surface-active ants forage no more than 5 m (Bruhl et al. 2003). Thus, in total, 180 points were sampled: 69 in primary forest, 13 in mature secondary forest, 36 in young secondary forest and 62 in cattle pasture, with sampling effort proportional to habitat cover. In general, arboreal ant species are not effectively sampled via pitfalls and our results, therefore, reflect surface-active ant species. Moreover, a single pitfall at each sampling point likely underestimated site-level species richness and probably better reflects differences between habitat types (i.e. pastures, secondary, and primary forest). Samples were collected at 24-h intervals across 4 days. Species were collected and identifications were made using reference materials from Instituto Alexander von Humboldt (IAvH) and Universidad Nacional, Colombia. Specimens were deposited in the IAvH collections based at Villa de Leyva, Colombia.

### Data analysis

We conducted all analyses using R version 3.1.1 (R Core Team 2013) except where specified otherwise. All tests used presence–absence data (i.e. incidence) as ants are social animals and so abundance measures may overestimate the presence of certain species (Woodcock et al. 2011). Because elevation negatively affects species richness in previous studies (Staab et al. 2014; Araújo and Fernandes 2003), elevation is taken into account in all models as a fixed effect.

### Species richness

To compare ant species richness between habitats, coverage-based rarefaction curves were used to control for different sample sizes (Chao and Chiu 2014). The ICE (Incidence-based Coverage Estimator) of species richness was used and can be interpreted as the fraction of the total incidence probabilities of the detected species in the reference sample (Chao and Chiu 2014). In calculating ICE, a cut-off value of *k *= 10 was used to separate species into “rare” and “abundant” groups.

At the sampling point level, generalised linear models (GLMs) with Poisson error and elevation as a fixed effect were used to test the relationship between habitat type and species richness and the relationship between secondary forest age and species richness. Model residuals were tested for spatial autocorrelation using a Moran’s *I* test (Ape package) and were found to have no effect (all *P* > 0.05), thus suggesting that land-use type explains the spatial structure of species richness (Edwards et al. 2014).

### Species composition

To compare species composition between habitats, non-metric multidimensional scaling (NMDS) ordination was employed, using the metaMDS function (Vegan package), with Bray–Curtis dissimilarity measure and 1000 permutations. Five primary forest sites from the upper elevational limit were removed as no species were recorded. Communities were standardised using the Wisconsin transformation (Vegan package), and species data were converted into a proportion of the total number of occurrences by site, therefore, accounting for differences among sites in the total number of ant occurrences (Woodcock et al. 2011). To test for differences in composition among habitat types, a permutational multivariate analysis of variance with 999 permutations, using the nonparametric ADONIS2 function (Vegan package), was utilised.

To assess potential changes in ant occurrence in response to habitat differences, we compared the occurrences of each species in primary forest against occurrences in secondary forests (summed young and mature) and pastures using GLMs with binomial errors (i.e. primary forest was the reference class).

A GLM with elevation as a fixed effect was used to test the relationship between Axis 1 of the NMDS and secondary forest age, as well as habitat type (primary forest, secondary forest and cattle pasture). We tested for spatial autocorrelation on community composition through a Mantel test (Vegan package), finding no effect (all *P* > 0.05).

### Primary forest species’ responses

To assess changes in species richness of primary forest species in response to habitat disturbance, we compared the occurrences of each species in primary forest against occurrences in secondary forests (summed young and mature) and pastures. We could not use a literature-based approach to define primary forest species because the majority of identifications were morpho-species. Therefore, we used the capture data to define primary forest species and focus the discussion. We defined a primary forest species as one with at least 1/2, 2/3 or 3/4 of its occurrences being in a primary forest site (excluding all species with a single occurrence). We present the results for three capture thresholds because any criteria are somewhat arbitrary and this enabled an assessment of how changing the criterion might impact the interpretation.

## Results

### Species richness

**Fig. 1:**
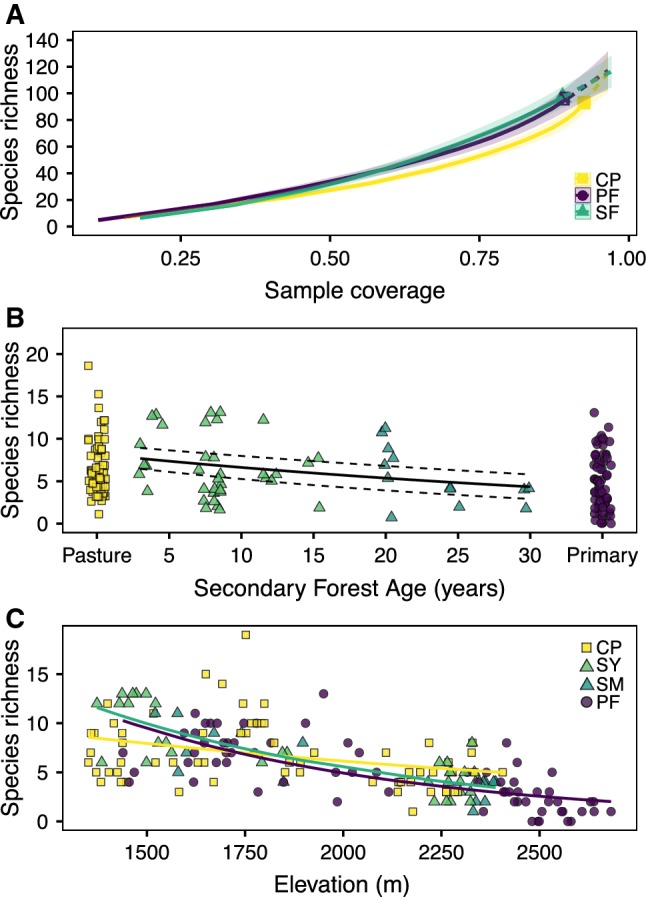
Observed species diversity of primary and secondary forest and pasture based on coverage-based rarefaction curves (**a**). Shading represents 95% confidence intervals. Dashed lines represent extrapolation results to the maximum coverage. Point-level species richness against habitat type with a linear regression of secondary forest age (**b**). Regression line plotted with predicted values from elevation model, with 95% confidence interval represented by dashed lines. Point-level species richness fit with generalised linear model (with Poisson errors) against elevation (m) for cattle pasture (CP), young (SY) and mature (SM) secondary, and primary (PF) forest (**c**)

Across the 180 points, 1072 occurrences of 153 ant species or morpho-species were sampled. Coverage-based species richness estimates for pastures, secondary forests, and primary forests were 0.93, 0.89, and 0.90, respectively, suggesting approximately equal sampling across habitat types (Table 1, Fig. 1a). A breakdown of total captures by functional group (Agosti et al. 2000) and habitat type is provided in Table 2.

**Table 1:** Summary of habitat-level species metrics across cattle pasture (CP), secondary forest (SF) and primary forest (CP)

Measure	CP	SF	PF
Occurrences	422	316	334
Observed species richness	93	97	96
Estimated species richness (ICE)	119.43	131.12	129.34
Sample coverage	92.51	88.92	89.69

**Table 2:** Summary of habitat-level functional group occurrences across cattle pasture (CP), secondary forest (SF) and primary forest (PF)

Functional group (*n*)	CP	SF	PF
Army (7)	6 (5)	10 (5)	5 (3)
Fungus cultivator (15)	33 (9)	14 (8)	38 (12)
Generalist (90)	231 (55)	177 (61)	186 (60)
Predatory (41)	152 (24)	115 (23)	105 (21)

Total numbers of species sampled are given in parentheses

Within secondary forests, there was a significant decline in species richness with increasing forest age (Fig. 1b; *β* = − 0.021, d*f* = 47, *z* = − 2.65, *P* < 0.01). This effect was, however, highly confounded by elevation (i.e. a significant positive relationship between age and elevation), with older secondary forests generally being at higher elevation (Fig. S2). When incorporating the effects of age and elevation, the relationship between species richness and forest age was not significant (*β* = − 0.005, d*f* = 46, *z* = − 0.64, *P* = 0.52). Forests (both primary and secondary) had the highest species richness values at low elevations, which declined significantly with increasing elevation (Fig. 1c; *β* = − 0.0012, d*f* = 47, *z* = − 7.70, *P* < 0.001 and *β* = − 0.0013, d*f* = 47, *z* = − 8.75, *P* < 0.001 for secondary and primary forests, respectively). The negative relationship between species richness and elevation, though still significant, was weaker for pastures, suggesting many species had already been lost (Fig. 1c; *β* = − 0.0005, d*f* = 47, *z* = − 3.48, *P* < 0.001).

### Species composition

**Fig. 2:**
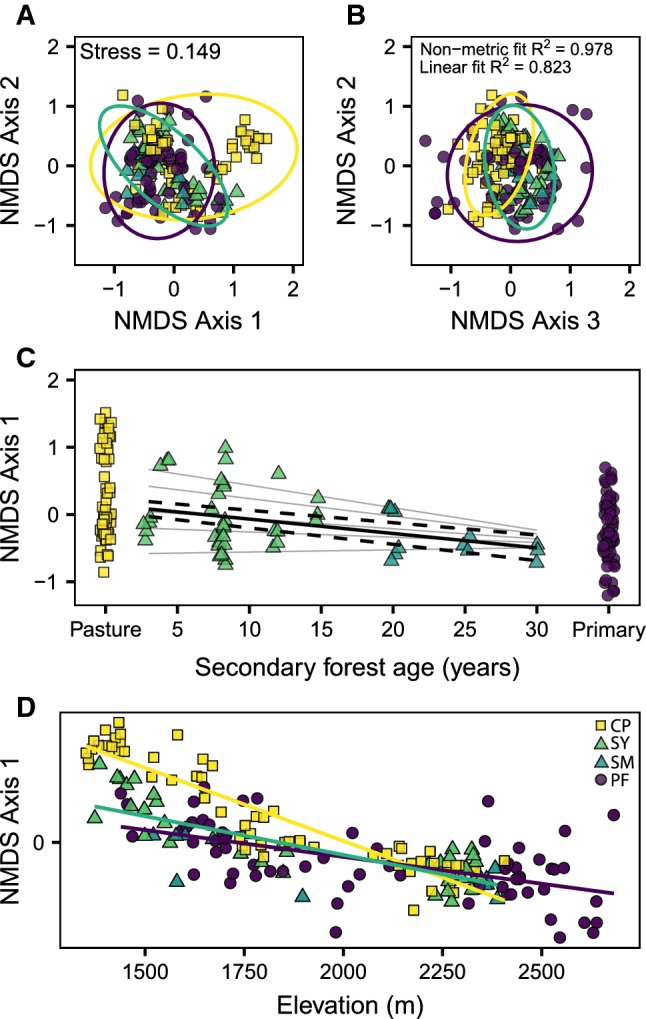
Non-metric multidimensional scaling (NMDS) ordination of standardised species composition within primary and secondary forest and pasture habitats (**a**, **b**). Ellipses represent 95% confidence intervals for each habitat type. NMDS axis 1 scores against habitat type with a linear regression of secondary forest age (**c**). Regression line plotted with predicted values from elevation model, with 95% confidence interval (CI) represented by dashed lines. Grey lines show fitted slopes for the interaction between secondary forest age and elevation, from low (top) to high (bottom) elevations. NMDS axis 1 scores plotted against elevation (m), by habitat type: cattle pasture (CP), young (SY) and mature (SM) secondary, and primary (PF) forest (**d**)

Ant species composition differed significantly between habitat types, with three NMDS axes required for sufficient community representations (*R*^2^ = 0.82, *F*_2,174_ = 5.42, *P* = 0.001, stress = 0.149; Fig. 2a, b). Relative to primary forests, more species increased in occurrence than decreased (Table S2), with nearly twice as many species showing a significantly higher occurrence in pastures (*n* = 11) than secondary forests (*n* = 6). Only three species decreased significantly in pastures and secondary forests, relative to primary forest (Table S2). This suggests many ant species were able to effectively exploit pastures and secondary forests. Two plots of changes in occurrence across habitat types and functional guilds can be found in the supplementary information (Figs. S3, S4).

There was a significant negative relationship between NMDS axis 1 and secondary forest age (Fig. 2c; *R*^2^ = 0.12, *β* = − 0.022, *F*_1,47_ = 6.52, *P* = 0.01). This relationship, however, depended on elevation (*R*^2^ = 0.70, *F*_3,45_ = 35.6, *P* < 0.001), with lower elevations having more negative slopes (Fig. 2c). The combined effect of age and elevation leads to large shifts in ant community composition (across NDMS Axis 1) in secondary forests at low elevations and little effect at high elevations (Fig. 2c).

There was a significant negative relationship between NMDS axis 1 and elevation (Fig. 2d; *R*^2^ = 0.59, *β* = − 0.001, *F*_1,173_ = 251.4, *P* < 0.001). This relationship, however, depended on habitat type (*R*^2^ = 0.75, *F*_5,169_ = 100.2, *P* < 0.001), with primary forest, secondary forests, and pastures having increasingly negative slopes (Fig. 2d). The combined effect of elevation and habitat type leads to the largest shift in ant community composition (across NDMS Axis 1) in pastures, followed by secondary and primary forests (Fig. 2d).

### Primary forest species’ responses

**Fig. 3:**
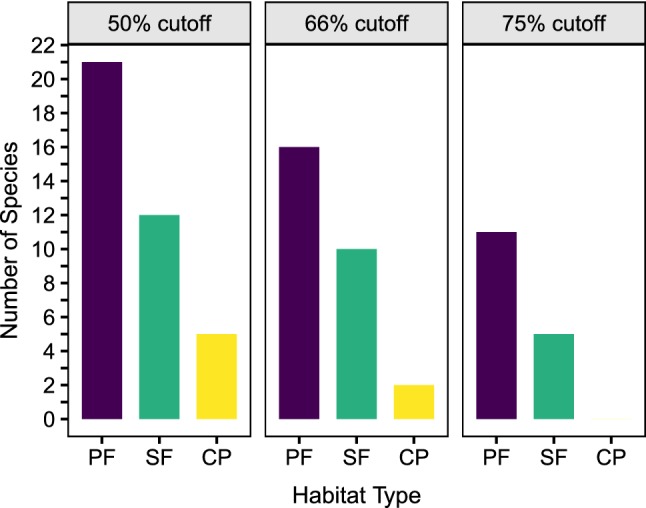
Summary of changes in occurrence of primary forest species in secondary forests (young and mature) and pastures. Results are displayed for three definitions of primary forest species, those having 1/2, 2/3, and 3/4 of their captures in primary forest

In general, pastures maintained few to no primary forest species, whereas secondary forests supported about half of the sampled richness (Fig. 3). The most stringent threshold (of 75% of records) for definition as a primary forest species revealed a wider gap between pasture and secondary forest. This suggests that although there was a significant shift in species composition, many primary forest ant species persisted in secondary forests.

## Discussion

There is an emerging global agenda of forest recovery, to reduce climate change, recover biodiversity and restore ecosystem services within degraded landscapes, with a global target to restore 350 million hectares by 2030 under the Bonn Challenge. Of particular importance are the montane tropics, where farming on oft marginal soils has rendered many ecosystems highly degraded, with little remaining natural habitat, and where there is already a phase of land abandonment (Nanni et al. 2019). We found that secondary forests, particularly those at low elevations, supported surface-active ant community composition and species richness similar to primary forest levels. Counter-intuitively, we also found that at higher elevations, pasturelands support slightly more surface-active ant species than either primary or secondary forest, owing to a less severe drop-off in species richness with elevation within pastures relative to forests. Thus, agriculture drives a significant reorganisation of elevation-diversity patterns of surface-active ants, potentially because pasturelands are warmer environments (Senior et al. 2017) making local conditions more favourable for some species that are in or near thermal debt at higher elevation.

Similar ant species richness within secondary versus primary forest echoes previous findings from the lowland and montane tropics (Online Resource Table 1; Hites et al. 2005; Chen et al. 2011), including findings for other study taxa (Hilt and Fiedler 2005; Gilroy et al. 2014a, b). Secondary forest sites harboured the highest observed and estimated total species richness, potentially driven by the mix of successional stages and associated habitat complexity (Rivera et al. 2013; Lassau and Hochuli 2004; Lassau et al. 2005). The higher richness in secondary forest may reflect a combination of pasture-adapted species alongside rapidly colonising forest species, because all secondary forest sites were adjacent to remnant primary forest (Gilroy and Edwards 2017). This may represent a source of ants for re-colonisation of recovering forest over time (Gilroy and Edwards 2017) and our results may overestimate the conservation value of newly recovering forests that are isolated from primary or mature secondary sources. However, in most tropical landscapes, secondary forests regrow next to contiguous patches of primary forest (Edwards et al. 2017).

In pasture habitats, a lack of tree cover is expected to result in the loss of species richness, given a positive relationship between tree cover and ant diversity in the Colombian Andes (Rivera et al. 2013). However, our study shows some ant species (about half) can persist even in highly degraded pasturelands, perhaps because many pastures within the study retained at least some scattered trees, hedgerows and forest patches (Feigl et al. 2006; Gilroy et al. 2014a, b; Schonberg et al. 2004) or because of source–sink dynamics from forest to pasture (Gilroy and Edwards 2017). In part, the high richness in pastures relative to forests could be explained by our sampling design. In general, pitfall traps under sample arboreal ant species (Agosti et al. 2000) and, therefore, our results on compositional changes better reflect ground-dwelling ants and are likely a conservative assessment of change, particularly for forested habitats.

We found that community composition varied across pasture, secondary and primary habitats. Common genera that persist at higher abundances in pasture than primary forest included *Solenopsis, Hypoponera,* and *Linepithema* species. Many of the species in these genera are considered generalist foragers that do well in the litter layer (Agosti et al. 2000; van Ingen et al. 2008). Yet, many taxa we found in primary forest still persisted in pasture, with some occurring at higher incidences, corroborating that many ant species can persist even in the most degraded pastures (Feigl et al. 2006), although there is the potential that such populations are in extinction debt. Importantly, 16 species of ground-dwelling ants were unique to primary forest, a total that we expect would have been higher if we had also sampled arboreal species and those engaged in interactions with myrmecophytic plants. Nevertheless, this highlights the irreplaceability of primary forests for vulnerable specialists (Barlow et al. 2007; Rivera et al. 2013). It also emphasises the question as to whether, and after how long, these species would establish in mature secondary forest.

This study is unique in revealing the impacts of secondary forest recovery on ants across a large elevational range of over 1300 m. Ant species richness and community diversity were both affected by elevation (this study; van Ingen et al. 2008; Longino and Colwell 2011; Staab et al. 2014; Tiede et al. 2017). We found abundance dropped towards zero above 2600 m, which is supported by similar findings from Ecuador (2800 m, Tiede et al. 2017), Costa Rica (2000 m, Longino and Colwell 2011), and central America (2500 m, Longino et al. 2014). This is likely driven by temperature (Longino and Colwell 2011; Longino et al. 2014) because mean annual temperature drops with greater vulnerability to fluctuations in unusually cold weather. Altitudinal declines in ant species richness are generally associated with the loss of tree cover at high elevations, as trees buffer temperature fluctuations and increase habitat heterogeneity (González del Pliego et al. 2016). Decreased habitat complexity, harsher habitat conditions, lower temperatures, reduced area and resources all, therefore, limit ant success at high elevation (Araújo and Fernandes 2003).

As ants are ectothermic with relatively small body sizes, they are highly sensitive to temperature fluctuations, particularly surface-active species (Staab et al. 2014; Araújo and Fernandes 2003). This is of particular importance to ants in the Andes, where global warming may require species to move to track narrow elevational distributions (Longino and Colwell 2011; Staab et al. 2014) and where simulations under RCP8.5 predict almost a tripling of ant species richness at higher elevations underpinned by dramatic changes in the abundance distribution of species, such that currently common species become very rare (Bishop et al. 2019). While we are unaware of studies documenting elevational movements in tropical ants as a result of climate change, highlighting a key knowledge gap, the average altitudes of 102 montane moth species in Borneo increased by 67 m between 1965 and 2007, with the 20 endemic species moving uphill by an average of 92 m (Chen et al. 2009). Interestingly, our results suggest that the effects of elevation on ant communities are less pronounced within cattle pasture habitats than forests, such that higher elevation pastures retain relatively high species diversity. This could be associated with the availability of suitable microclimates within relatively warm and open pastures, or related to the specific traits of ant species that thrive within pasture. The effects of land-use change on species’ elevational ranges are an area deserving of further study.

In conclusion, this study suggests that natural regenerating forests over the long term in the montane tropics will likely offer significant potential to harbour ant communities near primary forest levels, with secondary forests appearing to drive similar geographic patterns of ant richness with elevation as found in primary forest. Promoting their regeneration via land abandonment and their protection in the face of changing agricultural patterns, such as may occur in the post-peace settlement era within the Colombian Andes, is a conservation priority. It would offer substantial protection of ants and other taxa in the Andes (e.g. birds, dung beetles, amphibians; Gilroy et al. 2014a, b; Basham et al. 2016), as well as recovery and retention of important climate change-mitigating carbon stocks (Gilroy et al. 2014a, b).

## Electronic supplementary material

Below is the link to the electronic supplementary material.

Supplementary material 1 (DOCX 1519 kb)

## References

[CR1] Agosti D, Majer JD, Alonso LE, Schultz TR (2000). Ants: standard methods for measuring and monitoring biodiversity.

[CR2] Aide TM, Clark ML, Ricardo Grau H, Lopez-Carr D, Levy MA, Redo D, Bonilla-Moheno M, Riner G, Andrade-Nunez MJ, Muniz M (2013). Deforestation and reforestation of Latin America and the Caribbean (2001–2010). Biotropica.

[CR3] Araújo LM, Fernandes GW (2003). Altitudinal patterns in a tropical ant assemblage and variation in species richness between habitats. Lundiana.

[CR4] Barlow J, Gardner TA, Araujo IS, Ávila-Pires TC, Bonaldo AB, Costa JE, Esposito MC, Ferreira LV, Hawes J, Hernandez MI, Hoogmoed MS (2007). Quantifying the biodiversity value of tropical primary, secondary, and plantation forests. Proc Natl Acad Sci.

[CR5] Basham EW, González del Pliego P, Woodcock P, Medina CA, Gilroy JJ, Edwards DP (2016). Quantifying carbon and amphibian co-benefits from secondary forest regeneration in the Tropical Andes. Anim Conserv.

[CR7] Bihn JH, Gebauer G, Brandl R (2010). Loss of functional diversity of ant assemblages in secondary tropical forests. Ecology.

[CR8] Bishop TR, Parr CL, Gibb H, van Rensburg BJ, Braschler B, Chown SL, Foord SH, Lamy K, Munyai TC, Okey I, Tshivhandekano PG, Werenkraut V, Robertson MP (2019). Thermoregulatory traits combine with range shifts to alter the future of montane ant assemblages. Glob Change Biol.

[CR9] Bruhl CA, Eltz T, Linsenmair KE (2003). Size does matter: effects of tropical rainforest fragmentation on the leaf litter ant community in Sabah, Malaysia. Biodiv Conserv.

[CR10] Chao A, Chiu CH (2014) Species richness: estimation and comparison. Wiley StatsRef: Statistics Reference Online, pp 1–26

[CR12] Chen IC, Shiu HJ, Benedick S, Holloway JD, Chey VK, Barlow HS, Hill JK, Thomas CD (2009). Elevation increases in moth assemblages over 42 years on a tropical mountain. Proc Natl Acad Sci.

[CR13] Chen YQ, Li Q, Chen YL, Lu ZX, Zhou XY (2011). Ant diversity and bio-indicators in land management of lac insect agroecosystem in Southwestern China. Biodiv Conserv.

[CR17] Edwards DP, Magrach A, Woodcock P, Ji Y, Lim NTL, Edwards FA, Larsen TH, Hsu WW, Benedick S, Khen CV, Chung AYC, Reynolds G, Fisher B, Laurance WF, Wilcove DS, Hamer KC, Yu DW (2014). Selective-logging and oil palm: multitaxon impacts, biodiversity indicators, and trade-offs for conservation planning. Ecol Appl.

[CR18] Edwards DP, Massam MR, Haugaasen T, Gilroy JJ (2017). Tropical secondary forest regeneration conserves high levels of avian phylogenetic diversity. Biol Conserv.

[CR19] Feigl B, Cerri C, Piccolo M, Noronha N, Augusti K, Melillo J, Eschenbrenner V, Melo L (2006). Biological survey of a low-productivity pasture in Rondônia state, Brazil. Outlook Agr.

[CR20] Gilroy JJ, Edwards DP (2017). Source-sink dynamics: a neglected problem for landscape-scale biodiversity conservation. Curr Landsc Ecol Rep.

[CR21] Gilroy JJ, Edwards FA, Medina CA, Haugaasen T, Edwards DP (2014). Surrounding habitats mediate the trade-off between land-sharing and land-sparing agriculture in the tropics. J Appl Ecol.

[CR22] Gilroy JJ, Woodcock P, Edwards FA, Wheeler C, Baptiste BL, Uribe CAM, Haugaasen T, Edwards DP (2014). Cheap carbon and biodiversity co-benefits from forest regeneration in a hotspot of endemism. Nat Clim Change.

[CR23] González del Pliego P, Scheffers BR, Basham EW, Woodcock P, Wheeler CE, Gilroy JJ, Medina CA, Haugaasen T, Freckleton RP, Edwards DP (2016). Thermally buffered microhabitats recovery in tropical secondary forests following land abandonment. Biol Conserv.

[CR25] Guénard B, Weiser MD, Dunn RR (2012). Global models of ant diversity suggest regions where new discoveries are most likely are under disproportionate deforestation threat. Proc Natl Acad Sci.

[CR26] Hilt N, Fiedler K (2005). Diversity and composition of Arctiidae moth ensembles along a successional gradient in the Ecuadorian Andes. Divers Distrib.

[CR27] Hites NL, Mourão MA, Araújo FO, Melo MV, De Biseau JC, Quinet Y (2005). Diversity of the ground-dwelling ant fauna (Hymenoptera: formicidae) of a moist, montane forest of the semi-arid Brazilian “Nordeste”. Rev Biol Trop.

[CR28] Klimes P, Idigel C, Rimandai M, Fayle TM, Janda M, Weiblen GD, Novotny V (2012). Why are there more arboreal ant species in primary than in secondary tropical forests?. J Anim Ecol.

[CR29] Lamb D, Erskine PD, Parrotta JA (2005). Restoration of degraded tropical forest landscapes. Science.

[CR30] Lassau SA, Hochuli DF (2004). Effects of habitat complexity on ant assemblages. Ecography.

[CR31] Lassau SA, Cassis G, Flemons PK, Wilkie L, Hochuli DF (2005). Using high-resolution multi-spectral imagery to estimate habitat complexity in open-canopy forests: can we predict ant community patterns?. Ecography.

[CR32] Longino JT, Colwell RK (2011). Density compensation, species composition, and richness of ants on a neotropical elevational gradient. Ecosphere.

[CR33] Longino JT, Branstetter MG, Colwell RK (2014). How ants drop out: ant abundance on tropical mountains. PLoS One.

[CR34] Lopes CT, Vasconcelos HL (2008). Evaluation of three methods for sampling ground-dwelling ants in the Brazilian Cerrado. Neotrop Entomol.

[CR35] McCarthy DP, Donald PF, Scharlemann JPW, Buchanan GM, Balmford A, Green JMH, Bennun LA, Burgess ND, Fishpool LDC, Garnett ST, Leonard DL, Maloney RF, Morling P, Schaefer HM, Symes A, Wiedenfeld DA, Butchart SHM (2012). Financial costs of meeting global biodiversity conservation targets: current spending and unmet needs. Science.

[CR36] Nanni S, Sloan S, Aide M, Gressler J, Edwards DP, Grau C (2019). The neotropical reforestation hotspots: a biophysical and socioeconomic typology of contemporary forest expansion. Glob Environ Change.

[CR37] R Core Team (2013) R: a language and environment for statistical computing. R Foundation for Statistical Computing, Vienna

[CR38] Rivera LF, Armbrecht I, Calle Z (2013). Silvopastoral systems and ant diversity conservation in a cattle-dominated landscape of the Colombian Andes. Agr Ecosyst Environ.

[CR39] Schonberg LA, Longino JT, Nadkarni NM, Yanoviak SP, Gering JC (2004). Arboreal ant species richness in primary forest, secondary forest, and pasture habitats of a tropical montane landscape. Biotropica.

[CR40] Senior RA, Hill JK, González del Pliego P, Goode LK, Edwards DP (2017). A pantropical analysis of the impacts of forest degradation and conversion on local temperature. Ecol Evol.

[CR41] Staab M, Schuldt A, Assmann T, Bruelheide H, Klein AM (2014). Ant community structure during forest succession in a subtropical forest in South-East China. Acta Oecol.

[CR42] Stork NE, Srivastava DS, Eggleton P, Hodda M, Lawson G, Leakey RRB, Watt AD (2017). Consistency of effects of tropical-forest disturbance on species composition and richness relative to use of indicator taxa. Conserv Biol.

[CR43] Swinfield T, Afriandi R, Antoni F, Harrison RD (2016). Accelerating tropical forest restoration through the selective removal of pioneer species. For Ecol Manag.

[CR44] Tiede Y, Schlautmann J, Donoso DA, Wallis CIB, Bendix J, Brandl R, Farwig N (2017). Ants as indicators of environmental change and ecosystem processes. Ecol Indic.

[CR15] UNFCCC (2015). Adoption of the Paris agreement.

[CR45] van Ingen LT, Campos RI, Andersen AN (2008). Ant community structure along an extended rain forest–savanna gradient in tropical Australia. J Trop Ecol.

[CR46] Woodcock P, Edwards DP, Fayle TM, Newton RJ, Khen CV, Bottrell SH, Hamer KC (2011). The conservation value of South East Asia’s highly degraded forests: evidence from leaf-litter ants. Philos T Roy Soc B.

