# Supplementary material for: The impact of secondary forest regeneration on ground-dwelling ant communities in the Tropical Andes
Source: Oecologia. 2019 Sep 4;191(2):475–82. doi: 10.1007/s00442-019-04497-8 (PMC6763530; doi:10.1007/s00442-019-04497-8)
Supplement: Supplementary file 1 — Supplementary material 1 (DOCX 1519 kb) [file 442_2019_4497_MOESM1_ESM.docx]

**Supplementary Information**

**The impact of secondary forest regeneration on ground-dwelling ant communities in the Tropical Andes**

Matthew G. Hethcoat^1,2^*, Bethany J. King^2^*, Fernando Fernandez Castiblanco^3^, Claudia M. Ortiz-Sepúlveda^4^, Fabian Camilo Prada Achiardi^3^, Felicity A. Edwards^2^, Claudia Medina^5^, James J Gilroy^6^, Torbjørn Haugaasen^7^, David P Edwards^2^†

^1^School of Mathematics and Statistics, University of Sheffield, S3 7RH, UK

^2^Department of Animal and Plant Sciences, University of Sheffield, S10 2TN, UK

^3^Instituto de Ciencias Naturales Universidad Nacional, Carrera 30#45-03, Bogota, Colombia.

^4^UMR 8198 – Evolution, Ecologie et Paléontologie, CNRS, Université de Lille, 59000 Lille, France

^5^Instituto de Investigacion de Recursos Biologicos Alexander von Humboldt, Calle28A#15-09, Bogota, Colombia

^6^School of Environmental Science, University of East Anglia, Norwich NR4 7TJ, UK

^7^Faculty of Environmental Sciences and Natural Resource Management, Norwegian University of Life Sciences, Ås, Norway

**these authors contributed equally to this work*

†Correspondence: david.edwards@sheffield.ac.uk

**Contents:**

**Table S1.** Literature review and references.

**Table S2.** Species occurrences in each habitat with significant changes.

**Table S3.** Sample sizes from changes in occurrences of primary forest species found in pasture and secondary forests.

**Table S4.** Habitat pairwise comparisons for species richness and composition.

**Figure S1.** Map of study region.

**Figure S2.** Pairwise comparison of mean, habitat level elevation and relationship between forest age and elevation.

**Figure S3.** Treemap plot of the abundance of ant genera within cattle pastures (CP), primary forest (PF), and secondary forest (SF), subdivided by functional group.

**Figure S4.** Treemap plot of the abundance of ant genera functional groups, subdivided by their abundance within cattle pastures (CP), primary forest (PF), and secondary forest (SF).

**Table S1.** Literature review using WoS (Web of Science, search strings: ants OR formicidae AND regenerating forest* OR regrowth forest* OR secondary forest*, tropic*). Studies solely describing the fauna of a secondary forest (i.e. lacking a contrast with another land-use type), focusing on single ant species/genus, outside of the tropics, in grasslands, or utilising ‘secondary’ logged or degraded forests were excluded, to focus the review on ant community recovery in regenerating tropical forests on abandoned farmland. Where multiple studies used the same dataset, we only include the earliest published study. Studies included focused on either the entire ant community, or on a specific sub-group (i.e., leaf litter/ground or arboreal). Information was then extracted regarding geographical region, habitat contrasts (PF=primary forest), whether the study analysed above-ground carbon stocks (AGC), whether the study was lowland (<1,000 masl) or montane (>1,000 masl), and whether elevation was explicitly considered in analyses. Studies in the montane tropics are in **bold**.

| **Study** | **Geographic region** | **Habitat contrasts** | **Lowland/ Montane** | **Elevation** |
| --- | --- | --- | --- | --- |
| Belshaw & Bolton, 1993 | Ghana | Cocoa plantation, PF | Lowland | No |
| Bihn et al. 2008 | Atlantic forest, Brazil | Pasture, PF | Lowland | No |
| **Chen et al. 2011** | **SW China** | **Lac plantation, Corn** | **Montane** | **No** |
| de Castro Solar et al. 2016 | Amazon, Brazil | PF, Pasture, Mechanised agriculture, Silviculture | Lowland | No |
| Falcao et al. 2015 | Amazon, Brazil | Pasture, PF | Lowland | No |
| Feigl et al. 2006 | Amazon, Brazil | Pasture | Lowland | No |
| Floren & Linsenmair, 2005 | Sabah, Borneo | PF | Lowland | No |
| Gutierrez et al. 2016 | Amazon, Brazil | Fallows, Agroforestry | Lowland | No |
| Klimes et al. 2012 | New Guinea | PF | Lowland | No |
| Mathieu et al. 2005 | Amazon, Brazil | Pasture, PF | Lowland | No |
| Ottonetti et al. 2010 | Nicaragua | Shade coffee, Cocoa, Corn, Pasture | Lowland | No |
| Pacheco et al. 2009 | Atlantic Forest, Brazil | Pine plantation | Lowland | No |
| Roth et al. 1994 | Costa Rica | PF, Cacao, Banana | Lowland | No |
| **Schonberg et al. 2004** | **Costa Rica** | **Pasture, PF** | **Montane** | **No** |
| Silva et al. 2007 | Atlantic forest, Brazil | PF | Lowland | No |
| Suguituru et al. 2011 | Atlantic forest, Brazil | Eucalyptus plantation | Lowland | No |
| Takano et al. 2014 | Borneo | Fallows, PF | Lowland | No |
| Teodoro et al. 2010 | Coastal Ecuador | Coffee agroforestry | Lowland | No |
| **Tiede et al. 2017** | **Andes, Ecuador** | **PF** | **Montane** | **No** |
| Vasconcelos, 1999 | Amazon, Brazil | Pasture, PF | Lowland | No |
| Wilkie et al. 2009 | Amazon, Ecuador | PF | Lowland | No |
| Yeo et al. 2011 | Cote d’Ivoire | PF, plantations, fallows, crops, cocoa | Lowland | No |
| Zelikova & Breed, 2008 | Costa Rica | Young Acacia, pasture | Lowland | No |

**References for Literature Review**

Belshaw, R. and Bolton, B., 1993. The effect of forest disturbance on the leaf litter ant fauna in Ghana. *Biodiversity and Conservation*, ***2*(**6), pp.656-666.

Bihn, J.H., Verhaagh, M., Brändle, M. and Brandl, R., 2008. Do secondary forests act as refuges for old growth forest animals? Recovery of ant diversity in the Atlantic forest of Brazil. *Biological Conservation*, ***141***(3), pp.733-743.

Chen, Y.Q., Li, Q., Chen, Y.L., Lu, Z.X. and Zhou, X.Y., 2011. Ant diversity and bio-indicators in land management of lac insect agroecosystem in Southwestern China. *Biodiversity and Conservation*, ***20***(13), pp.3017-3038.

de Castro Solar, R.R., Barlow, J., Andersen, A.N., Schoereder, J.H., Berenguer, E., Ferreira, J.N. and Gardner, T.A., 2016. Biodiversity consequences of land-use change and forest disturbance in the Amazon: a multi-scale assessment using ant communities. *Biological Conservation*, ***197***, pp.98-107.

Falcão, J.C., Dáttilo, W. and Izzo, T.J., 2015. Efficiency of different planted forests in recovering biodiversity and ecological interactions in Brazilian Amazon. *Forest Ecology and Management*, ***339***, pp.105-111.

Feigl, B., Cerri, C., Piccolo, M., Noronha, N., Augusti, K., Melillo, J., Eschenbrenner, V. and Melo, L., 2006. Biological survey of a low-productivity pasture in Rondônia state, Brazil. *Outlook on Agriculture*, ***35***(3), pp.199-208.

Floren, A. and Linsenmair, K.E., 2005. The importance of primary tropical rain forest for species diversity: an investigation using arboreal ants as an example. *Ecosystems*, ***8***(5), pp.559-567.

Gutiérrez, J.A.M., Roussea, G.X., Andrade-Silva, J. and Delabie, J.H.C., 2016. Ants’ higher taxa as surrogates of species richness in a chronosequence of fallows, old-grown forests and agroforestry systems in the Eastern Amazon, Brazil. *Revista de Biología Tropical*, ***65***(1), pp.279-291.

Klimes, P., Idigel, C., Rimandai, M., Fayle, T.M., Janda, M., Weiblen, G.D. and Novotny, V., 2012. Why are there more arboreal ant species in primary than in secondary tropical forests? *Journal of Animal Ecology*, ***81***(5), pp.1103-1112.

Mathieu, J., Rossi, J.P., Mora, P., Lavelle, P., Martins, P.D.S., Rouland, C. and Grimaldi, M., 2005. Recovery of soil macrofauna communities after forest clearance in Eastern Amazonia, Brazil. *Conservation Biology*, ***19***(5), pp.1598-1605.

Ottonetti, L., Tucci, L., Frizzi, F., Chelazzi, G. and Santini, G., 2010. Changes in ground-foraging ant assemblages along a disturbance gradient in a tropical agricultural landscape. *Ethology, Ecology & Evolution*, ***22***(1), pp.73-86.

Pacheco, R., Silva, R.R., Morini, M.S.D.C. and Brandão, C.R., 2009. A comparison of the leaf-litter ant fauna in a secondary atlantic forest with an adjacent pine plantation in Southeastern Brazil. *Neotropical Entomology*, ***38***(1), pp.55-65.

Roth, D.S., Perfecto, I. and Rathcke, B., 1994. The effects of management systems on ground‐foraging ant diversity in Costa Rica. *Ecological Applications*, **4**(3), pp.423-436.

Schonberg, L.A., Longino, J.T., Nadkarni, N.M., Yanoviak, S.P. and Gering, J.C., 2004. Arboreal ant species richness in primary forest, secondary forest, and pasture habitats of a tropical montane landscape. *Biotropica*, ***36***(3), pp.402-409.

Silva, R.R., Feitosa, R.S.M. and Eberhardt, F., 2007. Reduced ant diversity along a habitat regeneration gradient in the southern Brazilian Atlantic Forest. *Forest Ecology and Management*, ***240***(1), pp.61-69.

Suguituru, S.S., Silva, R.R., Souza, D.R.D., Munhae, C.D.B. and Morini, M.S.D.C., 2011. Ant community richness and composition across a gradient from Eucalyptus plantations to secondary Atlantic Forest. *Biota Neotropica*, ***11***(1), pp.369-376.

Takano, K.T., Nakagawa, M., Itioka, T., Kishimoto-Yamada, K., Yamashita, S., Tanaka, H.O., Fukuda, D., Nagamasu, H., Ichikawa, M., Kato, Y. and Momose, K., 2014. The Extent of Biodiversity Recovery During Reforestation After Swidden Cultivation and the Impacts of Land-Use Changes on the Biodiversity of a Tropical Rainforest Region in Borneo. In *Social-Ecological Systems in Transition* (pp. 27-49). Springer Japan.

Teodoro, A.V., Sousa-Souto, L., Klein, A.M. and Tscharntke, T., 2010. Seasonal contrasts in the response of coffee ants to agroforestry shade-tree management. *Environmental Entomology*, ***39***(6), pp.1744-1750.

Tiede Y, Schlautmann J, Donoso DA, Wallis CIB, Bendix J, Brandl R, Farwig N (2017) Ants as indicators of environmental change and ecosystem processes. *Ecological Indicators* 83:527-537.

Vasconcelos, H.L., 1999. Effects of forest disturbance on the structure of ground-foraging ant communities in central Amazonia. *Biodiversity and Conservation*, ***8***(3), pp.407-418.

Wilkie, K.T.R., Mertl, A.L. and Traniello, J.F., 2009. Diversity of ground-dwelling ants (Hymenoptera: Formicidae) in primary and secondary forests in Amazonian Ecuador. *Myrmecological News*, ***12***, pp.139-147.

Yeo, K., Konate, S., Tiho, S. and Camara, S.K., 2011. Impacts of land use types on ant communities in a tropical forest margin (Oum Cte dIvoire). *African Journal of Agricultural Research*, ***6***(1), pp.260-274.

Zelikova, T.J. and Breed, M.D., 2008. Effects of habitat disturbance on ant community composition and seed dispersal by ants in a tropical dry forest in Costa Rica. *Journal of Tropical Ecology*, ***24***(03), pp.309-316.

**Table S2:** Ant occurrences for all species sampled in primary forest (PF), mature (SM) and young (SY) secondary forest and cattle pasture (CP). Asterisks indicate significant changes in occurrences compared with primary forest at the *p* < 0.1*, *p* < 0.05**, and *p* < 0.01*** levels. Note the statistics refer to secondary forest collectively (i.e. summed counts from SM and SY).

|  | | | | |
| --- | --- | --- | --- | --- |
| **Species** | **PF** | **SM** | **SY** | **CP** |
| *Acanthoponera minor* | 0 | 1 | 0 | 0 |
| *Acanthoponera mucronata* | 0 | 0 | 1 | 0 |
| *Acromyrmex aspersus* | 1 | 0 | 0 | 5 |
| *Acromyrmex landolti* | 0 | 0 | 0 | 1 |
| *Anochetus bispinosus* | 1 | 0 | 0 | 0 |
| *Anochetus* sp. 1 | 1 | 0 | 0 | 6^*^ |
| *Aphaenogaster* sp.1 | 0 | 0 | 2 | 0 |
| *Aphaenogaster* sp. 2 | 1 | 0 | 0 | 5 |
| *Apterostigma* sp. 1 | 2 | 0 | 0 | 0 |
| *Apterostigma* sp. 2 | 0 | 0 | 1 | 0 |
| *Apterostigma* sp. 3 | 9 | 1 | 1 | 0 |
| *Apterostigma* sp. 4 | 5 | 0 | 0 | 4 |
| *Apterostigma* sp. 5 | 3 | 1 | 0 | 9^*^ |
| *Apterostigma* sp. 6 | 4 | 0 | 0 | 4 |
| *Atta cephalotes* | 0 | 0 | 2 | 4 |
| *Brachymyrmex* sp. 1 | 0 | 0 | 1 | 1 |
| *Brachymyrmex* sp. 2 | 0 | 0 | 0 | 5 |
| *Brachymyrmex* sp. 3 | 0 | 0 | 0 | 2 |
| *Camponotus* sp. 1 | 0 | 0 | 2 | 0 |
| *Camponotus* sp. 2 | 1 | 0 | 3 | 1 |
| *Camponotus* sp. 3 | 0 | 0 | 0 | 2 |
| *Chelyomyrmex morosus* | 0 | 0 | 0 | 1 |
| *Chyphomyrmex* sp. 1 | 1 | 0 | 0 | 1 |
| *Crematogaster* sp. 1 | 0 | 0 | 0 | 1 |
| *Cyphomyrmex cornotus* | 1 | 0 | 1 | 0 |
| *Cyphomyrmex podargus* | 1 | 0 | 0 | 0 |
| *Cyphomyrmex* sp. 1 | 2 | 0 | 2 | 2 |
| *Cyphomyrmex* sp. 2 | 7 | 1 | 3 | 3 |
| *Dolichoderus* sp. 1 | 0 | 0 | 0 | 1 |
| *Eciton rapax* | 0 | 0 | 2 | 0 |
| *Ectatomma ruidum* | 0 | 0 | 0 | 3 |
| *Ectatomma tuberculatum* | 0 | 0 | 0 | 13 |
| *Gnamptogenys bisulca* | 7 | 3 | 0 | 0 |
| *Gnamptogenys perspicax* | 1 | 0 | 0 | 0 |
| *Gnamptogenys striatula* | 36 | 12 | 25^**†^ | 28 |
| *Hypoponera* sp. 1 | 0 | 0 | 0 | 1 |
| *Hypoponera* sp. 1 | 1 | 0 | 0 | 0 |
| *Hypoponera* sp. 2 | 1 | 0 | 0 | 7^**^ |
| *Labidus coecus* | 1 | 0 | 3 | 2 |
| *Labidus mars* | 3 | 0 | 2 | 1 |
| *Labidus praedator* | 1 | 0 | 1 | 1 |
| *Leptogenys* sp. 1 | 4 | 1 | 1 | 0 |
| *Linepithema* sp. 1 | 5 | 0 | 3 | 17^***^ |
| *Linepithema* sp. 2 | 2 | 0 | 0 | 11^**^ |
| *Megalomyrmex* sp. 1 | 0 | 0 | 0 | 1 |
| *Neivamyrmex* sp. 1 | 0 | 2 | 0 | 0 |
| *Nomamyrmex* sp. 1 | 0 | 0 | 0 | 1 |
| *Nylanderia* sp. 1 | 2 | 0 | 2 | 0 |
| *Nylanderia* sp. 2 | 9 | 0 | 3 | 4 |
| *Nylanderia* sp. 3 | 3 | 0 | 2 | 5 |
| *Nylanderia* sp. 4 | 5 | 1 | 3 | 7 |
| *Nylanderia* sp. 5 | 5 | 0 | 0 | 0 |
| *Nylanderia* sp. 6 | 1 | 1 | 3 | 6^*^ |
| *Octostruma balzani* | 1 | 0 | 1 | 5 |
| *Octostruma impressa* | 0 | 0 | 2 | 0 |
| *Octostruma inca* | 0 | 0 | 2 | 3 |
| *Octostruma stenoscapa* | 3 | 0 | 0 | 0 |
| *Odontomachus affinis* | 5 | 1 | 3 | 6 |
| *Odontomachus erythrocephalus* | 0 | 0 | 1 | 23 |
| *Odontomachus opaciventris* | 0 | 0 | 1 | 0 |
| *Odontomachus* sp. 1 | 2 | 0 | 0 | 0 |
| *Pachycondyla aenescens* | 12 | 4 | 18^***†^ | 15 |
| *Pachycondyla apicalis* | 2 | 0 | 1 | 0 |
| *Pachycondyla becculata* | 0 | 0 | 0 | 1 |
| *Pachycondyla carbonaria* | 9 | 4 | 9^*†^ | 6 |
| *Pachycondyla chyzeri* | 11 | 3 | 6 | 2^**^ |
| *Pachycondyla ferruginea* | 0 | 0 | 1 | 0 |
| *Pachycondyla harpax* | 1 | 0 | 2 | 18^***^ |
| *Pachycondyla holcotyle* | 0 | 0 | 0 | 2 |
| *Pachycondyla impressa* | 0 | 0 | 4 | 5 |
| *Pachycondyla purpurascens* | 0 | 0 | 1 | 0 |
| *Pachycondyla* sp. 1 | 0 | 0 | 0 | 1 |
| *Pheidole* sp. 1 | 2 | 2 | 0 | 0 |
| *Pheidole* sp. 2 | 1 | 2 | 1 | 0 |
| *Pheidole* sp. 3 | 5 | 0 | 1 | 0 |
| *Pheidole* sp. 4 | 0 | 0 | 3 | 4 |
| *Pheidole* sp. 5 | 2 | 0 | 0 | 14^***^ |
| *Pheidole* sp. 6 | 1 | 0 | 0 | 5 |
| *Pheidole* sp. 7 | 3 | 1 | 7^**†^ | 2 |
| *Pheidole* sp. 8 | 3 | 2 | 1 | 1 |
| *Pheidole* sp. 9 | 4 | 0 | 1 | 5 |
| *Pheidole* sp. 10 | 0 | 0 | 3 | 0 |
| *Pheidole* sp. 11 | 7 | 1 | 8 | 3 |
| *Pheidole* sp. 12 | 1 | 0 | 3 | 4 |
| *Pheidole* sp. 13 | 3 | 0 | 2 | 0 |
| *Pheidole* sp. 14 | 0 | 3 | 4 | 2 |
| *Pheidole* sp. 15 | 0 | 0 | 0 | 1 |
| *Pheidole* sp. 16 | 1 | 0 | 0 | 0 |
| *Pheidole* sp. 17 | 0 | 0 | 1 | 0 |
| *Pheidole* sp. 18 | 1 | 0 | 1 | 0 |
| *Pheidole* sp. 19 | 1 | 0 | 0 | 0 |
| *Pheidole* sp. 20 | 4 | 1 | 3 | 0 |
| *Pheidole* sp. 21 | 1 | 0 | 1 | 0 |
| *Pheidole* sp. 22 | 0 | 1 | 0 | 1 |
| *Pheidole* sp. 23 | 2 | 2 | 0 | 0 |
| *Pheidole* sp. 24 | 0 | 0 | 1 | 0 |
| *Pheidole* sp. 25 | 9 | 2 | 10 | 6 |
| *Pheidole* sp. 26 | 2 | 2 | 6^**†^ | 0 |
| *Pheidole* sp. 27 | 1 | 2 | 5^**†^ | 1 |
| *Pheidole* sp. 28 | 3 | 0 | 3 | 1 |
| *Pheidole* sp. 29 | 0 | 0 | 1 | 0 |
| *Pheidole* sp. 30 | 3 | 1 | 1 | 1 |
| *Pheidole* sp. 31 | 0 | 0 | 2 | 0 |
| *Pheidole* sp. 32 | 0 | 0 | 2 | 2 |
| *Pheidole* sp. 33 | 1 | 0 | 0 | 0 |
| *Pheidole* sp. 34 | 4 | 1 | 0 | 4 |
| *Pheidole* sp. 35 | 8 | 1 | 0^*†^ | 0 |
| *Pheidole* sp. 36 | 1 | 0 | 1 | 1 |
| *Pheidole* sp. 37 | 0 | 0 | 1 | 0 |
| *Pheidole* sp. 38 | 6 | 1 | 1 | 1 |
| *Pheidole* sp. 39 | 7 | 1 | 0 | 0 |
| *Pheidole* sp. 40 | 0 | 0 | 1 | 0 |
| *Pheidole* sp. 41 | 3 | 1 | 0 | 0 |
| *Pheidole* sp. 42 | 8 | 3 | 4 | 1^*^ |
| *Pheidole* sp. 43 | 2 | 0 | 0 | 0 |
| *Pheidole* sp. 44 | 4 | 0 | 0 | 0 |
| *Pheidole* sp. 45 | 5 | 1 | 1 | 3 |
| *Pheidole* sp. 46 | 3 | 0 | 0 | 2 |
| *Pheidole* sp. 47 | 2 | 0 | 4 | 0 |
| *Pheidole* sp. 49 | 1 | 0 | 0 | 0 |
| *Pheidole* sp. 50 | 0 | 0 | 0 | 1 |
| *Pheidole* sp. 51 | 1 | 0 | 0 | 0 |
| *Procryptocerus rudis* | 1 | 0 | 1 | 0 |
| *Pseudomyrmex* sp. 1 | 0 | 0 | 0 | 1 |
| *Pyramica* sp. 1 | 2 | 0 | 0 | 3 |
| *Pyramica* sp. 2 | 0 | 0 | 0 | 1 |
| *Rhopalothrix* sp. 1 | 2 | 0 | 2 | 0 |
| *Rhopalothrix* sp. 2 | 0 | 0 | 1 | 0 |
| *Rogeria cuneola* | 2 | 0 | 0 | 1 |
| *Simopelta* sp. 1 | 0 | 1 | 0 | 0 |
| *Solenopsis* sp. 1 | 1 | 0 | 1 | 5 |
| *Solenopsis* sp. 2 | 0 | 0 | 0 | 2 |
| *Solenopsis* sp. 3 | 0 | 1 | 3 | 4 |
| *Solenopsis* sp. 4 | 2 | 0 | 5 | 3 |
| *Solenopsis* sp. 5 | 6 | 1 | 3 | 7 |
| *Solenopsis* sp. 6 | 2 | 1 | 2 | 8^**^ |
| *Solenopsis* sp. 7 | 2 | 0 | 2 | 9^**^ |
| *Solenopsis* sp. 8 | 8 | 0 | 4 | 19^***^ |
| *Solenopsis* sp. 9 | 7 | 0 | 4 | 4 |
| *Solenopsis* sp. 10 | 0 | 0 | 0 | 1 |
| *Solenopsis* sp. 11 | 0 | 0 | 1 | 17 |
| *Solenopsis* sp. 12 | 1 | 0 | 0 | 1 |
| *Stenamma* sp. 1 | 0 | 1 | 0 | 0 |
| *Strumigenys louisianae* | 0 | 0 | 0 | 3 |
| *Strumigenys* sp. 1 | 3 | 1 | 3 | 0 |
| *Strumigenys* sp. 2 | 1 | 0 | 0 | 2 |
| *Strumigenys* sp. 3 | 1 | 0 | 0 | 1 |
| *Strumigenys* sp. 4 | 0 | 0 | 0 | 1 |
| *Strumigenys* sp. 5 | 0 | 0 | 0 | 1 |
| *Strumigenys* sp. 6 | 1 | 0 | 0 | 0 |
| *Trachymyrmex* sp. 1 | 2 | 0 | 1 | 0 |
| *Typhlomyrmex pusillus* | 1 | 0 | 1 | 3 |
| *Wasmannia auropunctata* | 0 | 0 | 4 | 6 |

^†^ statistic is for secondary forest and includes summed counts for SM and SY

**Table S3:** Summary statistics for the pairwise comparisons of species richness and community composition between habitat types

|  | **Species richness** | | **Composition** | |
| --- | --- | --- | --- | --- |
| **Habitat comparison** | ***t*-value** | **Significance (*P*)** | ***F*-value** | **Significance (*P*)** |
| Primary – Pasture | 3.330 | 0.003 | 6.143 | 0.001 |
| Primary – Secondary | 2.487 | 0.036 | 2.908 | 0.002 |
| Secondary – Pasture | -0.618 | 0.810 | 7.201 | 0.001 |

**STUDY SITES:**


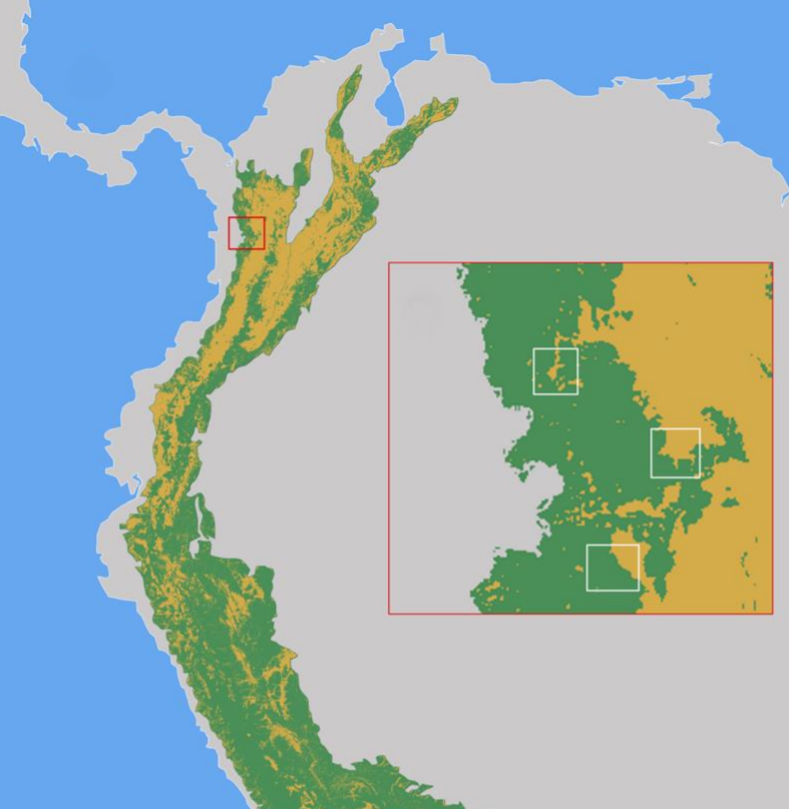


**Figure S1.** Adapted from Gilroy et al. (2014). Map of study landscape, showing the extent of habitat conversion in the Tropical Andes with red box indicating study region and the study sites within, denoted by white boxes. Colours indicate land use type; existing natural vegetation (dark green) and agriculture or other uses (pale orange).

**THE EFFECT OF ELEVATION:**

Like previous research, elevation was found to significantly reduce species richness ( *p* < 0.001). The mean transect elevation significantly different between all three habitats, with primary transects on average nearly 300 m higher than pasture transects (Fig. S2). Moreover there was a significant positive relationship between forest age and elevation – with older forests generally being at higher elevations.


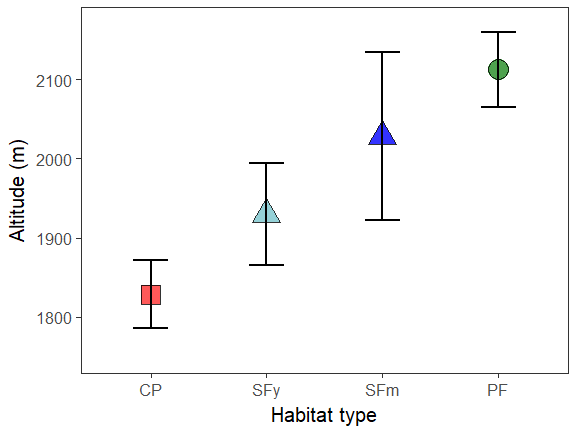

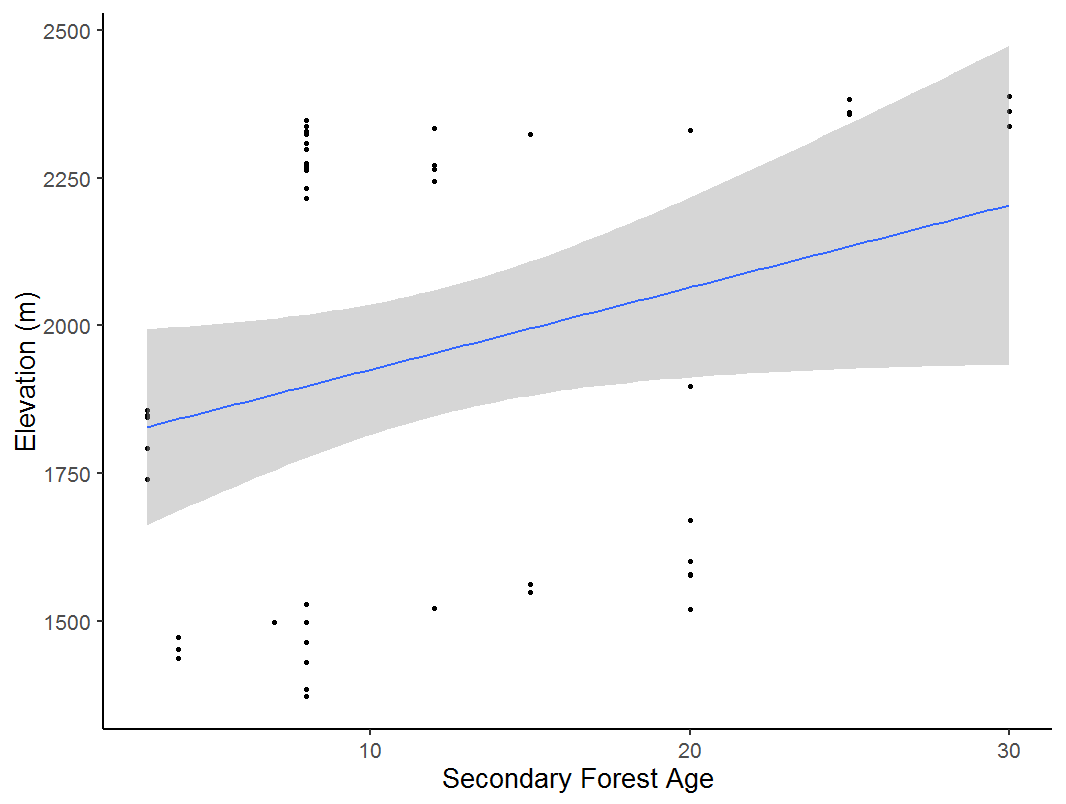


**A**

**B**

**Figure S2.** Pairwise comparison of mean, habitat level elevation; cattle pasture (CP), secondary (young and mature) and primary forest (PF), with standard error bars (A). Relationship between secondary forest age and elevation was significant (B).

**B**


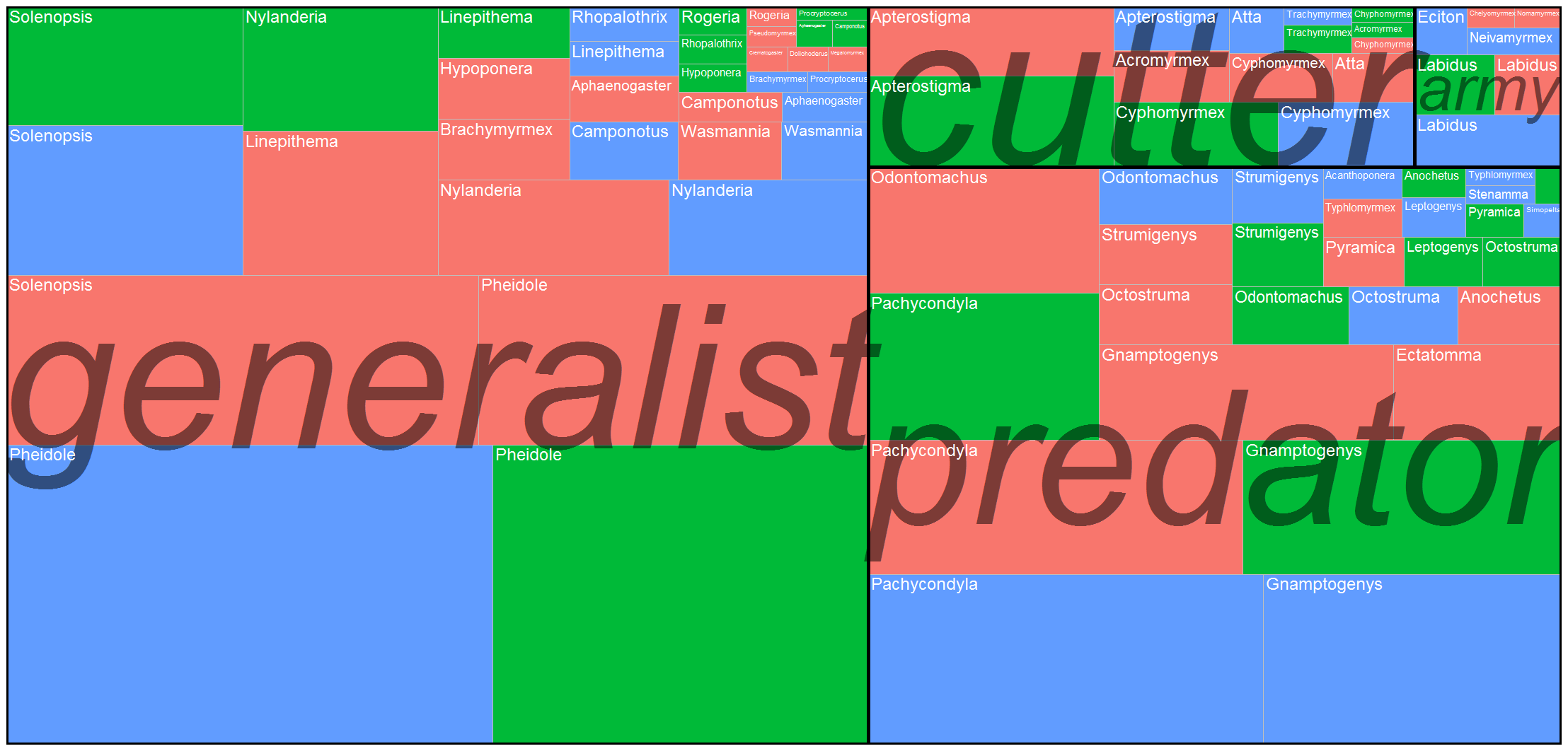

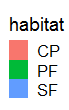


**Figure S3.** Treemap plot of the abundance of ant genera within cattle pastures (CP, red), primary forest (PF, green), and secondary forest (SF, blue). Treemap is further divided by the functional group those genera belong to (generalist, predator, cutter, and army). Data are scaled to reflect sampling effort and represent summed occurrences per 100 pitfalls sampled.


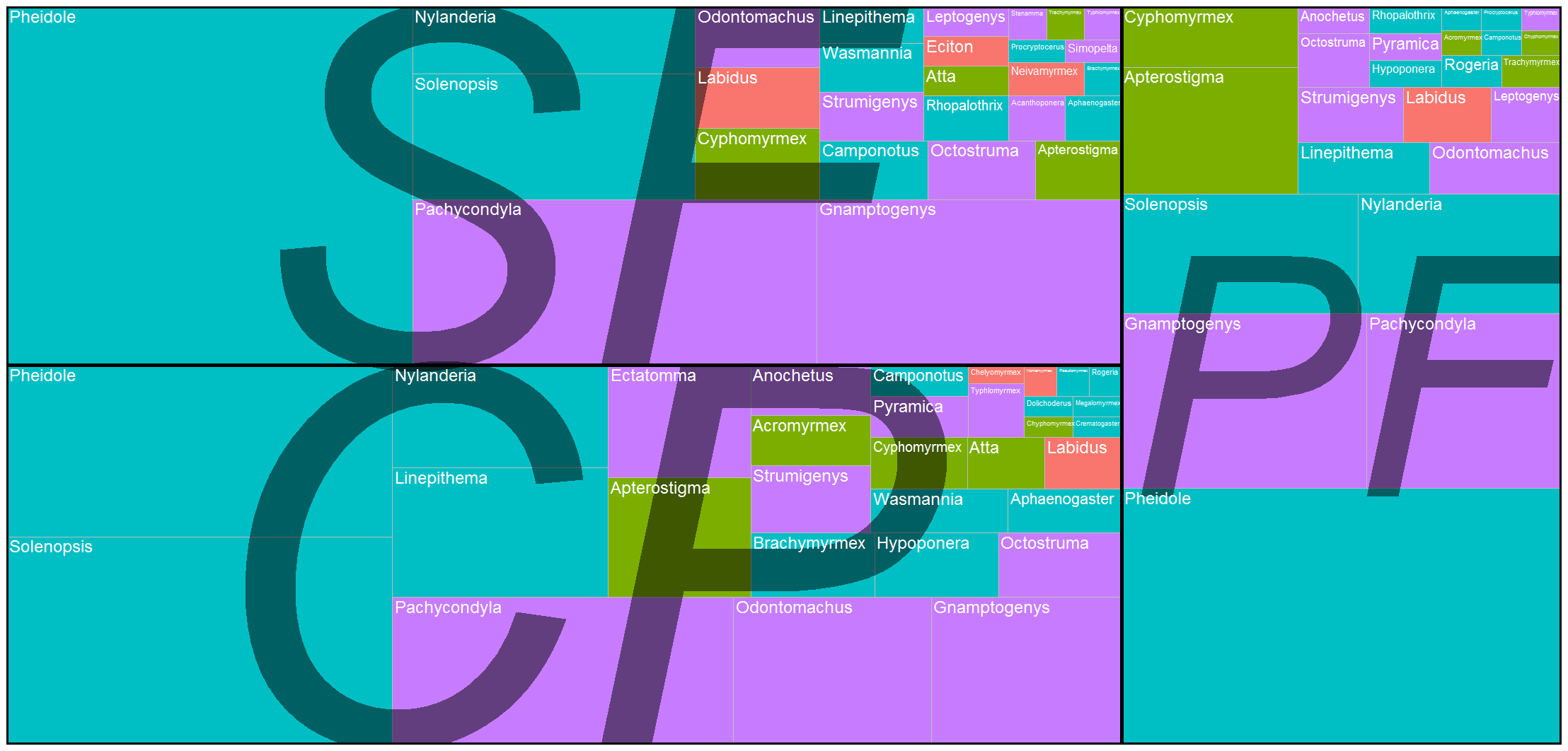

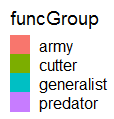


**Figure S4.** Treemap plot of the abundance of ant genera within cattle pastures (CP), primary forest (PF), and secondary forest (SF). The treemap plot is further divided by the functional group each genus belongs to (predator, generalist, cutter, and army). Data are scaled to reflect sampling effort and represent summed occurrences per 100 pitfalls sampled.
